# Deep brain stimulation as neuroimmune-metabolic modulation in Parkinson’s disease and beyond: tissue response, glial remodeling, and disease modification

**DOI:** 10.3389/fimmu.2026.1940474

**Published:** 2026-10-07

**Authors:** Hao Deng, Xiaolong Li, Dihu Wang, Youheng Peng, Maoyu Wang, Hao Liang, Yang Wu, Linglong Xiao, Wei Pan, Wei Wang, Senlin Yin

**Affiliations:** 1 Department of Neurosurgery, Gaoxin Hospital Affiliated to West China Second School of Medicine, Sichuan University, Chengdu, Sichuan, China; 2 Department of Emergency Medicine, West China Hospital, Sichuan University, Chengdu, Sichuan, China; 3 Southwest Medical University, Luzhou, Sichuan, China; 4 Department of Neurosurgery, West China Hospital, Sichuan University, Chengdu, Sichuan, China; 5 Department of Neurosurgery, The First Affiliated Hospital of Sun Yat-sen University, Guangzhou, Guangdong, China

**Keywords:** deep brain stimulation, electrode-tissue interface, neuroimmune modulation, neuroinflammation, Parkinson’s disease

## Abstract

Deep brain stimulation (DBS) is an established symptomatic therapy for Parkinson’s disease (PD), yet its biological effects extend beyond modulation of abnormal basal ganglia circuitry. This review conceptualizes DBS in PD as a neuroimmune-metabolic intervention acting across the electrode-tissue interface, glial networks, peripheral immunity, and activity-dependent metabolic systems. PD is characterized by convergent immune and metabolic vulnerability. These processes create a disease environment in which electrical stimulation may interact with inflammation, excitotoxicity, bioenergetic stress, and neuronal resilience. We distinguish implantation-related tissue responses from stimulation-induced biological remodeling. Electrode insertion produces acute injury, blood-brain barrier (BBB) disruption, peri-electrode edema, gliosis, and foreign-body responses, which should not be conflated with the effects of chronic stimulation. Active DBS may modulate central cytokine expression, microglial and astrocytic phenotypes, inflammasome activity, astrocytic glutamate transport, and brain-peripheral immune crosstalk. Emerging evidence also suggests a neurometabolic dimension, including changes in regional glucose utilization, PD-related metabolic network expression, oxidative stress, and neurotrophic signaling. However, these effects are context-dependent and influenced by multiple factors. Although preclinical studies support potential neuroprotective mechanisms involving BDNF/TrkB signaling, mitophagy, reduced excitotoxic-inflammatory coupling, and immune-metabolic rebalancing, clinical evidence remains insufficient to establish DBS as a disease-modifying therapy. Future studies should integrate multiple methods such as longitudinal imaging, electrophysiology, immune profiling and metabolomics. DBS should be understood as a symptomatic circuit-based therapy that may also interact with neuroimmune and metabolic processes, while current evidence remains insufficient to establish a disease-modifying role in PD.

## 1 Introduction

Deep brain stimulation (DBS) is an established neurosurgical therapy for Parkinson’s disease (PD), particularly for patients with motor fluctuations, medication-refractory tremor, dyskinesia, or advanced motor complications. Randomized clinical trials have shown that DBS can improve motor function and quality of life compared with best medical therapy in appropriately selected patients (1, 2). Traditionally, the therapeutic effects of DBS have been interpreted mainly through electrophysiological mechanisms, including modulation of pathological firing patterns, disruption of abnormal oscillatory activity, alteration of synaptic transmission, and reorganization of circuit-level dynamics within the cortico-basal ganglia-thalamic network (3, 4). More recently, connectomic DBS studies have extended this view by showing that clinical effects of subthalamic nucleus (STN) DBS in PD are related to structural and functional connectivity profiles and to modulation of distributed brain networks rather than the stimulated nucleus alone (5, 6). However, a purely circuit-centered interpretation does not fully capture the biological consequences of chronically implanted and actively stimulated electrodes. Neural activity is energetically expensive, and changes in firing patterns, synaptic transmission, and network synchronization are inevitably coupled to changes in glucose utilization, mitochondrial ATP production, neurotransmitter cycling, and glial-neuronal metabolic support. Molecular imaging studies further indicate that DBS can reshape regional glucose metabolism and PD-related metabolic network expression, suggesting that stimulation acts on distributed functional-metabolic systems rather than on a circumscribed electrophysiological node alone (7–9).

Accumulating evidence therefore suggests that DBS should also be considered as a biological intervention acting at the interface between neural activity, tissue response, immune regulation, and metabolic homeostasis. DBS electrodes are foreign bodies implanted into living brain tissue, and their placement induces acute and chronic local responses, including mechanical injury, blood-brain barrier (BBB) disruption, peri-electrode edema, gliosis, microglial activation, and variable fibrosis (10, 11). These implantation-related responses are distinct from, but may interact with, the effects of electrical stimulation. Therefore, a careful analysis of DBS-associated neuroimmune effects must separate the consequences of electrode insertion from those of active stimulation. This distinction is particularly important in PD, because the disease itself is characterized by an interdependent neuroimmune and metabolic landscape. Central neuroinflammatory processes involving microglia and astrocytes are increasingly recognized as contributors to dopaminergic vulnerability, α-synuclein propagation, oxidative stress, and synaptic dysfunction (12–14). In parallel, mitochondrial dysfunction, impaired PINK1/Parkin-dependent mitochondrial quality control, oxidative-redox imbalance, and lipid-lysosomal abnormalities are core features of PD biology and can amplify inflammatory signaling through mitochondrial damage-associated molecular patterns, inflammasome activation, lipid mediators, and impaired α-synuclein clearance (15–19). Thus, DBS is introduced into a disease environment already shaped by glial dysfunction, immune dysregulation, mitochondrial stress, and altered lipid and energy metabolism.

Recent studies have begun to connect DBS with immune modulation and metabolic remodeling more directly. Clinical and experimental data suggest that DBS may alter inflammatory markers, glial activation, cytokine expression, peripheral immune-cell profiles, and neuroinflammatory signaling in PD models and patients (17, 20, 21). Notably, DBS has been reported to halt PD-related immune dysregulation in brain and peripheral blood, while STN stimulation in animal models may reduce striatal cytokine expression, inflammasome reactivity, and excitotoxic-inflammatory coupling (20, 21). At the same time, clinically patterned stimulation can selectively induce inflammatory mediators in human central nervous system (CNS) cells *in vitro*, underscoring that DBS is not simply anti-inflammatory in all contexts (22). Emerging evidence also supports a metabolic dimension of DBS action: STN-DBS has been associated with changes in cerebral glucose metabolism, metabolic network topology, and, in experimental and translational studies, mitigation of oxidative stress through mitophagy-related mechanisms (7–9, 23). These findings provide a plausible bridge between electrical neuromodulation, glial immune state, mitochondrial quality control, and neuronal resilience.

This review therefore focuses on the neuroimmune and neurometabolic effects of DBS in PD. By distinguishing tissue responses from stimulation-induced immune and metabolic remodeling, this review aims to clarify whether DBS may function not only as a symptomatic circuit therapy but also as a modulator of neuroimmune-metabolic homeostasis and a potential contributor to neuroprotection in PD.

## 2 Immune and metabolic landscape of Parkinson’s disease

### 2.1 Central and peripheral immune dysregulation

PD is increasingly recognized as a neuroimmune disorder in which inflammatory processes interact with α-synuclein pathology, mitochondrial dysfunction, oxidative stress, and dopaminergic neuronal vulnerability. Although degeneration of nigrostriatal dopaminergic neurons remains the central pathological feature, immune activation is not merely a secondary response to neuronal death. Instead, converging evidence suggest that both central and peripheral immune mechanisms participate in disease initiation, propagation, and progression (12, 14). The immune and metabolic landscape of PD is illustrated in **Figure 1**.

**Figure 1:**
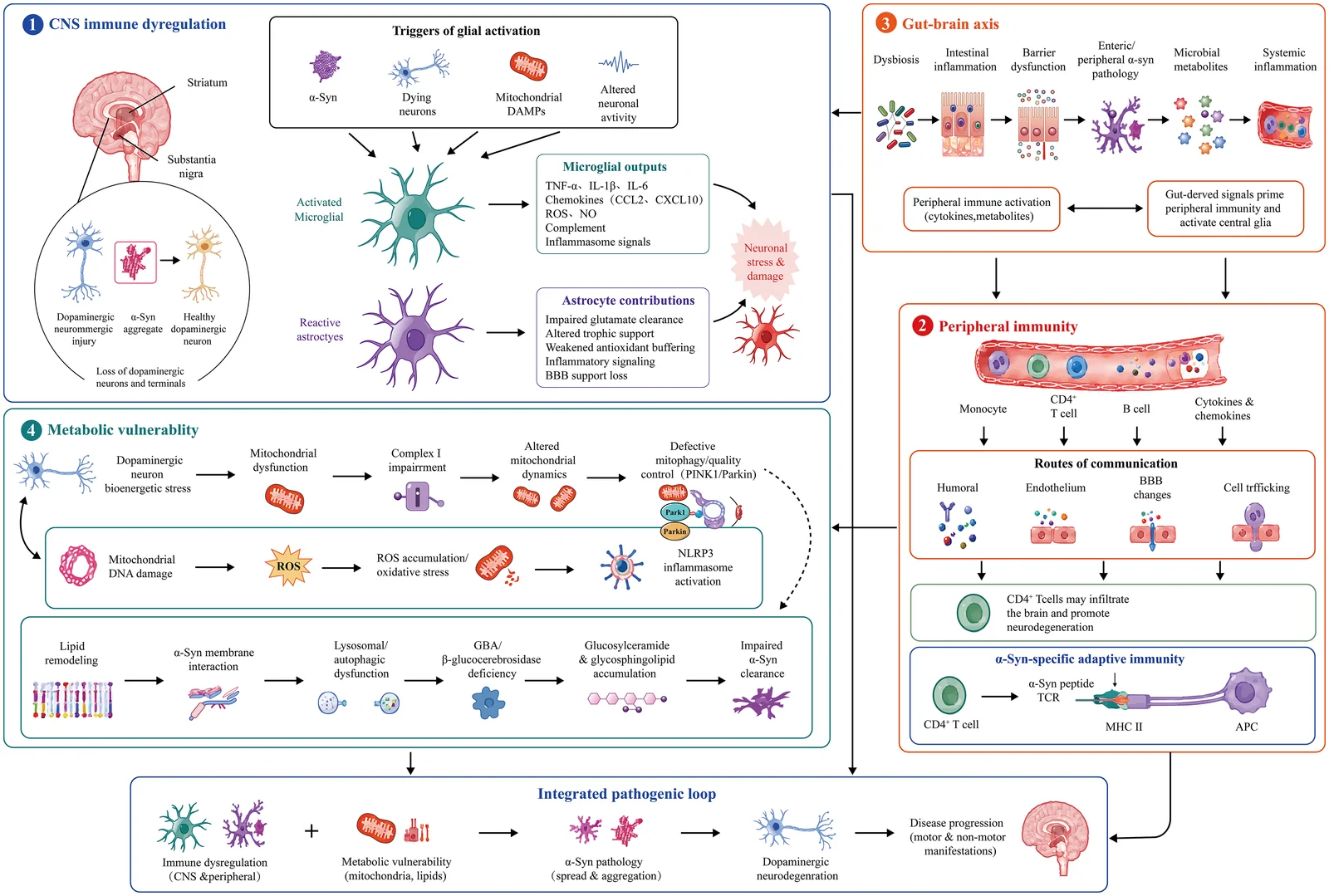
Neuroimmune-metabolic landscape of PD. This schematic illustrates the interconnected immune and metabolic mechanisms driving PD progression. In the central nervous system, pathological α-synuclein accumulation, mitochondrial damage-associated signals, and altered neuronal activity activate microglia and astrocytes, resulting in inflammatory mediator release, impaired homeostasis, and neuronal stress. Peripheral immune dysregulation, including cytokine signaling, immune-cell trafficking, and α-synuclein-specific adaptive immunity, further contributes to neuroinflammatory propagation. The gut–brain axis links microbiome alterations, intestinal dysfunction, and systemic inflammation with central immune activation. Metabolic vulnerability interacts with immune pathways to amplify α-synuclein pathology. Together, these processes form an integrated pathogenic loop connecting neuroimmune dysregulation, metabolic disturbance, dopaminergic neurodegeneration, and PD progression. To improve conceptual readability, individual mediators are grouped into broader categories such as inflammatory mediators, glial dysfunction, metabolic vulnerability, and gut-derived signals.

Within the central nervous system, microglia and astrocytes are the principal mediators of neuroinflammation. Microglia can be activated by misfolded α-synuclein, dying neurons, mitochondrial damage-associated signals, and altered neuronal activity. Once activated, they may release pro-inflammatory cytokines, chemokines, reactive oxygen species, nitric oxide, complement-related mediators, and inflammasome-associated signals that amplify local neuronal stress (12, 14). Astrocytes also contribute substantially to the PD immune landscape. Under physiological conditions, astrocytes support neurotransmitter recycling, metabolic homeostasis, antioxidant defense, and BBB integrity. In PD, however, dysfunctional or reactive astrocytes may impair glutamate clearance, alter trophic support, weaken antioxidant buffering, and reinforce inflammatory signaling (12, 13). Thus, microglia and astrocytes form a dynamic inflammatory network that can be protective or damaging depending on disease stage, regional context, and activation state.

Peripheral immunity is also increasingly implicated in PD. Immune responses appear to be early and dynamic rather than restricted to advanced neurodegeneration (24). Peripheral immune alterations may include changes in monocytes, T-cell subsets, B cells, cytokines, and chemokines, with evidence that systemic inflammation can communicate with the brain through humoral routes, endothelial activation, BBB changes, autonomic pathways, and immune-cell trafficking (25–27). In experimental PD, infiltration of CD4+ lymphocytes into the brain contributes to dopaminergic neurodegeneration, supporting a direct pathogenic role for adaptive immunity (28). In patients, T cells can recognize α-synuclein-derived peptides, providing evidence for antigen-specific autoimmune-like responses in PD (29, 30). The balance between pro-inflammatory and regulatory T-cell responses is another important component. Th17-related immune activity may promote neuroinflammatory injury, whereas regulatory T cells can restrain excessive inflammation. In a preclinical PD model, regulatory T cells attenuated Th17-mediated nigrostriatal dopaminergic neurodegeneration, suggesting that immune balance rather than global immune suppression may be critical for neuroprotection (31).

Finally, the gut-brain axis provides a systemic framework for PD-related immune activation. Gastrointestinal dysfunction, microbiome alterations, intestinal inflammation, and α-synuclein pathology in enteric or peripheral tissues may contribute to immune priming and neuroinflammatory propagation (32, 33). Microbial metabolites and intestinal barrier dysfunction may influence systemic inflammation and, indirectly, central glial activation. Therefore, the immune landscape of PD should be viewed as a multi-compartment process linking brain-resident glia, peripheral immune cells, α-synuclein-specific adaptive responses, and gut-derived inflammatory signals.

### 2.2 Metabolic vulnerability in PD

Metabolic vulnerability is a central feature of PD pathobiology and provides an important bridge between neurodegeneration and neuroinflammation. Dopaminergic neurons in the substantia nigra pars compacta are especially susceptible to bioenergetic stress because they have extensive axonal arborization, autonomous pacemaking activity, high calcium handling demands, and dopamine oxidation chemistry that can increase oxidative burden. These features impose sustained mitochondrial demand and make these neurons particularly vulnerable to mitochondrial defects and reactive oxygen species (ROS) accumulation (34).

Mitochondrial dysfunction in PD involves multiple convergent mechanisms, including complex I impairment, altered mitochondrial dynamics, defective mitophagy, impaired mitochondrial quality control, and mitochondrial DNA damage (16, 18, 35–38). Genetic evidence strongly implicates PINK1 and Parkin in mitochondrial quality control, and disruption of these pathways can compromise the removal of damaged mitochondria, thereby increasing neuronal vulnerability (18, 35). Mitochondrial dysfunction also intersects with inflammation: damaged mitochondria may release mitochondrial damage-associated molecular patterns, promote oxidative stress, and amplify Toll-like receptor and NLRP3 inflammasome-dependent signaling (37, 38). Thus, oxidative stress should not be viewed merely as a downstream consequence of inflammation. Instead, redox imbalance, mitochondrial damage, inflammasome activation, and microglial priming may form a self-reinforcing pathogenic loop.

Lipid and lysosomal pathways represent another major metabolic axis in PD. α-Synuclein binds lipid membranes, and changes in membrane lipids, sphingolipids, cholesterol-related domains, and vesicular lipid composition can influence α-synuclein conformation, oligomerization, aggregation, and synaptic vesicle trafficking (15). GBA-related glucocerebrosidase deficiency further links PD to lysosomal dysfunction, glucosylceramide and glucosyl sphingosine accumulation, impaired autophagic-lysosomal clearance, α-synuclein aggregation, mitochondrial stress, and neuroinflammation (19, 39). Therefore, the metabolic landscape of PD extends beyond ATP generation to include redox homeostasis, mitochondrial quality control, lipid remodeling, lysosomal-autophagic function, and α-synuclein handling.

### 2.3 Immunometabolic coupling in PD

The immune and metabolic abnormalities of PD are closely coupled at the cellular level. Activated microglia undergo immunometabolic reprogramming, often involving increased glycolytic flux, mitochondrial remodeling, altered oxidative phosphorylation, and enhanced production of ROS and nitric oxide. These metabolic shifts can sustain cytokine production, inflammasome activation, and chronic inflammatory tone (37, 40). Conversely, mitochondrial injury and oxidative stress can further prime microglia, creating a feed-forward interaction between metabolic failure and neuroinflammation. This bidirectional coupling is central to the framework of this review: mitochondrial stress and redox imbalance can prime microglial inflammatory responses, whereas glial inflammatory activation can further compromise neuronal bioenergetics and astrocytic metabolic support.

Astrocytes also contribute to immunometabolic regulation in PD. Beyond their inflammatory roles, astrocytes regulate extracellular glutamate clearance, glutamate-glutamine cycling, lactate-mediated neuronal energy support, glutathione-dependent antioxidant defense, and BBB metabolic integrity (41–43). Dysfunction of these astrocytic functions may strengthen the link between excitotoxicity, oxidative stress, impaired neuronal energy supply, and inflammatory signaling.

Peripheral immune cells are similarly shaped by metabolic state. T-cell differentiation, including the balance between pro-inflammatory Th17 responses and regulatory T cells, is influenced by glycolysis, mitochondrial fitness, lipid metabolism, and redox status. This is relevant for PD because peripheral immune dysregulation, α-synuclein-specific T-cell responses, and systemic inflammatory signals may interact with central glial activation (25, 29–31). Stimulation-related immune changes may reflect not only direct neuromodulatory effects but also secondary changes in motor activity, dopaminergic medication, autonomic tone, and systemic metabolism. PD should be conceptualized as a neuroimmune-metabolic disorder.

## 3 Tissue responses to electrode implantation

### 3.1 Acute insertion-related tissue injury and peri-electrode edema

DBS electrode implantation inevitably produces a local mechanical injury before any chronic stimulation effect occurs. The electrode trajectory disrupts neural parenchyma, small vessels, extracellular matrix, and the BBB, initiating an acute wound-healing response that includes edema, serum protein extravasation, activation of resident microglia, recruitment of blood-derived immune cells, and early astrocytic reactivity (10, 11). This early response is clinically important because it may transiently affect local excitability, impedance, and postoperative neurological symptoms. It is also mechanistically important because it forms the biological background upon which chronic stimulation subsequently acts. The dynamic tissue responses following DBS electrode implantation were presented in **Figure 2**.

**Figure 2:**
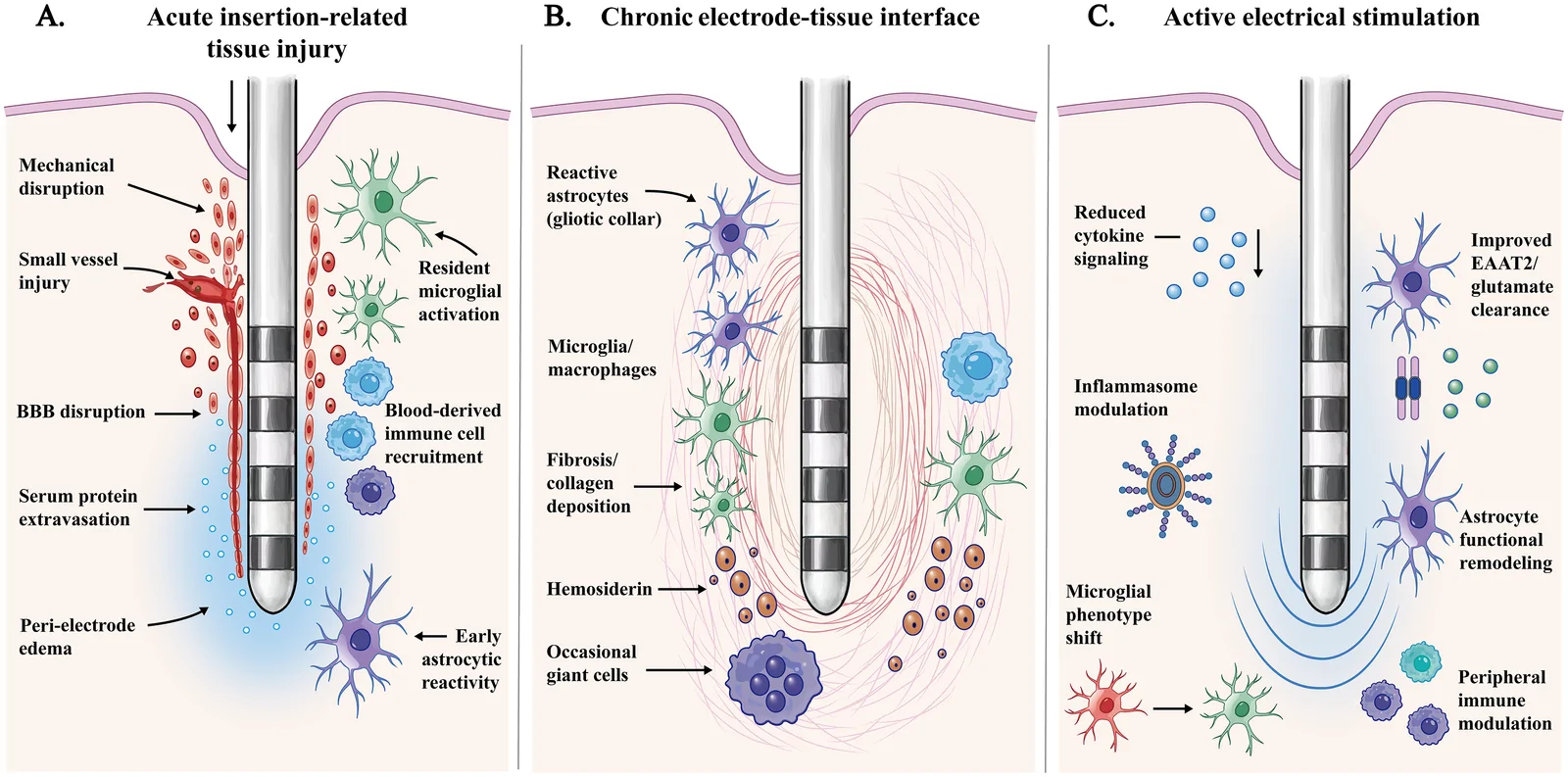
Dynamic tissue responses following DBS electrode implantation and stimulation. DBS implantation triggers a dynamic tissue response involving acute injury, chronic foreign body reaction, and stimulation-dependent neuroimmune remodeling. **(A)** Acute insertion-related tissue injury. Electrode penetration disrupts neural tissue, small vessels, and the BBB, leading to edema, serum protein extravasation, microglial activation, immune-cell recruitment, and early astrocytic reactivity. **(B)** Chronic electrode–tissue interface. Long-term implantation induces localized gliosis, microglia/macrophage accumulation, fibrosis, collagen deposition, hemosiderin accumulation, and occasional giant cell formation, shaping the peri-electrode microenvironment. **(C)** Active electrical stimulation. Chronic stimulation modulates cytokine signaling, inflammasome activity, glial phenotypes, astrocytic function, glutamate homeostasis, and peripheral immune responses. These processes highlight the DBS electrode–brain interface as a dynamic biological-electrical system in which implantation-related responses and stimulation-induced neuroimmune effects coexist but remain mechanistically distinct.

Peri-electrode edema is one of the best-described acute-to-subacute manifestations of this implantation response. In a retrospective clinical series, Saitoh et al. reported postoperative FLAIR/T2 hyperintensities around DBS electrodes in patients with PD, emphasizing that non-infectious peri-electrode edema can occur without neurological deficits and may involve different radiographic patterns, including deep white matter abnormalities and more extensive superficial white matter edema (44). A prospective MRI study by Borellini et al. found peri-lead edema in all examined patients after DBS surgery for PD, suggesting that this phenomenon may be more common than previously appreciated when systematic postoperative MRI is performed (45). Other reports have described dramatic radiographic abnormalities with peri-electrode edema and contrast enhancement in the absence of infection or hemorrhage, occasionally accompanied by seizures or other clinical symptoms (46).

The time course of edema also supports its interpretation as a dynamic inflammatory or wound-healing phenomenon rather than a static surgical lesion. Nolt et al. prospectively evaluated postoperative MRI changes and showed that white matter edema associated with DBS leads evolves over time after implantation (47). These observations suggest that peri-electrode edema represents a spectrum ranging from subclinical imaging change to symptomatic inflammatory reaction. This acute response should be clearly separated from stimulation-induced neuroimmune modulation. Failure to distinguish implantation-related inflammation from stimulation-related effects may lead to overinterpretation of DBS as either pro-inflammatory or anti-inflammatory. Importantly, acute insertion-related injury should also be interpreted as a local metabolic stress response. BBB disruption, vasogenic edema, tissue deformation, ionic disequilibrium, local hypoxia-like stress, ROS generation, and mitochondrial stress may coexist with early inflammatory signaling around the electrode trajectory. These processes may contribute not only to postoperative imaging abnormalities but also to transient changes in extracellular conductivity and electrode impedance. Therefore, the early peri-electrode environment should be viewed as an inflammatory-metabolic wound-healing niche rather than a purely structural lesion (11, 48).

### 3.2 Chronic foreign body response

After the acute insertion phase, the implanted DBS lead becomes a chronic foreign body within the brain. The long-term tissue response is typically characterized by a peri-electrode capsule or collar composed of reactive astrocytes, microglia/macrophages, variable fibrous elements, hemosiderin deposition, and occasional multinucleated giant cells. This response resembles the general biological reaction to neural implants, but DBS electrodes are larger and deeper than many microelectrode systems, making the electrode-tissue interface particularly relevant for both safety and stimulation efficacy (49, 50).

Postmortem studies provide direct evidence of chronic tissue remodeling around DBS leads. Vedam-Mai et al. quantitatively examined gliosis around distal DBS lead tips in postmortem cases and found tissue reaction and astrocytic responses around the lead, with a mean gliotic collar measured by H&E and GFAP staining in the range of approximately 100–160 μm (10). Significant fibrosis and prominent microglial response were uncommon but present in selected cases, suggesting that most chronic DBS leads are associated with limited but measurable gliosis, while exaggerated fibrotic or inflammatory reactions may represent rare complications (10). In another postmortem report, the same group described prominent collagenous fibrosis around a DBS lead tip in a patient with PD, associated with focal hemosiderin deposition, chronic inflammation, and mild gliosis (51). This case illustrates that although severe fibrosis is unusual, chronic implant-associated immune and extracellular matrix responses can occasionally become substantial. Long-term clinicopathologic observations support a similar conclusion. Sun et al. reported postmortem findings after 71 months of STN-DBS for PD, describing a thin glial capsule, foreign-body giant cell reaction, piloid gliosis, and hemosiderin around the leads (52). A 12-year clinicopathologic follow-up in essential tremor likewise found electrode tracts rimmed by thin fibrous sheaths with multinucleated giant cells, reactive gliosis, and lymphocytic infiltration, but overall minimal foreign body reaction around chronically implanted electrodes (53). Pediatric postmortem data after bilateral GPi-DBS for status dystonicus also confirm that chronic DBS leads can induce localized histopathological tissue reactions (54).

Overall, the chronic response to DBS electrodes appears spatially restricted in most cases, but it is biologically meaningful. Astrocytic encapsulation, microglial activation, fibrosis, and inflammatory cell infiltration may alter the local extracellular environment and shape current distribution. These changes are especially relevant when interpreting long-term DBS efficacy, impedance evolution, and possible immune consequences of chronic neuromodulation. From a metabolic perspective, chronic gliosis and fibrotic encapsulation may alter more than local histology. The peri-electrode capsule can modify extracellular matrix composition, ionic diffusion, fluid exchange, nutrient delivery, metabolite clearance, and the effective electrical conductivity of the surrounding tissue. Glial scarring should therefore not be regarded as a static barrier. Rather, it may represent a persistent immunometabolic interface in which astrocytes, microglia/macrophages, extracellular matrix remodeling, and local redox state continue to shape both tissue homeostasis and current spread (50, 51, 54).

### 3.3 Human postmortem evidence and its interpretive limitations

Human postmortem studies are indispensable for understanding DBS-associated tissue responses because they directly reveal the cellular and structural consequences of long-term implantation. However, they also have important limitations: sample sizes are small, disease backgrounds vary, stimulation targets differ, lead durations are heterogeneous, and tissue processing can distort the electrode tract. Therefore, postmortem findings should be interpreted as complementary to animal models and imaging studies rather than as a complete map of the living electrode-brain interface.

A systematic review of postmortem studies in PD emphasized that DBS lead tracks are generally associated with localized gliosis, macrophage or giant cell responses, hemosiderin deposition, and variable fibrosis, while widespread destructive tissue injury is not typically observed (55). This supports the clinical impression that DBS is generally compatible with long-term brain implantation. Nevertheless, the same body of evidence shows that chronic leads are not biologically inert. Vedam-Mai et al. demonstrated measurable gliosis around lead tips in a postmortem cohort, and rare cases showed marked fibrosis or microglial response (10). Sun et al. observed a thin glial capsule and foreign-body giant cell reaction around both active and inactive contacts, suggesting that at least part of the response reflects the implanted material and surgical tract rather than electrical stimulation alone (52). Postmortem studies also reveal disease- and duration-specific complexity. In essential tremor, 12 years of DBS was associated with thin fibrous sheaths, multinucleated giant cells, reactive gliosis, and lymphocytic infiltration, but the reaction was described as relatively limited (53). In pediatric dystonia, postmortem histopathology after GPi-DBS provided additional evidence that chronic leads can produce localized glial and inflammatory changes across different disease contexts (54). These observations indicate that tissue response is not unique to PD, although PD-related neuroinflammation may influence how the surrounding brain reacts to implantation.

The main limitation is that postmortem tissue reflects an endpoint. It cannot fully resolve the temporal sequence from acute edema to chronic encapsulation, nor can it reliably distinguish surgical injury, passive foreign body response, stimulation effects, and terminal disease processes.

### 3.4 Electrode-tissue interface, impedance, and functional consequences

The electrode-tissue interface is not only a histological phenomenon; it is also an electrical and therapeutic determinant. DBS depends on current delivery through the contact surface into surrounding brain tissue. Any change in the peri-electrode space, encapsulation layer, extracellular fluid composition, or tissue conductivity can alter impedance and reshape the volume of tissue activated. Thus, tissue responses induced by implantation may influence both stimulation efficiency and clinical programming.

Computational and clinical studies support this concept. Yousif et al. modeled the peri-electrode space and showed that the electrode-brain interface can significantly affect current distribution during DBS (56). Their work highlighted that structural and biophysical features around the electrode, including pathological changes such as giant cell formation or altered peri-electrode space, may influence stimulation fields. Satzer et al. later demonstrated that DBS electrode impedance varies with anatomical contact location in patients with PD, indicating that local tissue environment contributes to electrical properties relevant to stimulation delivery (57). These findings connect tissue biology directly to device performance.

Recent reviews have further emphasized that the DBS electrode is an “active” interface embedded in living tissue. Evers and Lowery reviewed evidence showing that impedance commonly increases in the weeks after implantation and stabilizes over several months, while stimulated electrodes may show different impedance behavior than non-stimulated electrodes (58). Evers et al. also investigated stimulation-induced changes at the electrode-tissue interface in rats and concluded that chronic stimulation can influence interface properties, with potential effects on electric field distribution and neural activation (59). These results suggest that the interface is shaped by both implantation and stimulation, making it a dynamic biological-electrical system.

From a neuroimmune perspective, this has several implications. First, glial encapsulation and inflammatory remodeling may affect therapeutic thresholds by changing local conductivity. Second, the same tissue response that protects the brain from a foreign body may reduce efficient charge transfer. Third, immune or fibrotic responses may contribute to variability in clinical programming requirements across patients and over time. Finally, strategies that reduce excessive foreign body response, improve material biocompatibility, or stabilize the electrode-tissue interface may enhance long-term DBS performance (49, 50, 60). Impedance changes and electric field distribution can be understood as products of a coupled biophysical and tissue-metabolic interface. The DBS interface is not a passive boundary between metal and brain, but a living biological-electrical microenvironment in which tissue metabolism, immune activation, and stimulation physics jointly determine long-term device performance. Several tissue responses induced by DBS electrode implantation were summarized in **Table 1**.

**Table 1:** Tissue responses induced by DBS electrode implantation.

Category	Main tissue response	Key cellular/Molecular features	Evidence source
Acute insertion injury	Mechanical disruption of brain parenchyma	Local tissue damage, blood-brain barrier disruption, microvascular injury	Animal and neurohistopathological studies (9, 47, 48)
Peri-electrode edema	Transient edema along the DBS lead	T2/FLAIR hyperintensity, non-infectious inflammatory reaction, occasional contrast enhancement	Postoperative MRI studies and clinical reports (42–45)
Early glial activation	Local microglial and astrocytic response	Activated microglia, reactive astrocytes, cytokine release, wound-healing response	Animal studies and neural implant tissue-response literature (9, 47, 58)
Chronic foreign body response	Long-term tissue encapsulation around electrode	Gliotic collar, microglia/macrophages, multinucleated giant cells, collagen/fibrosis	Human postmortem and review studies (8, 9, 49–53)
Gliosis around lead tip	Localized astrocytic response	GFAP-positive astrocytic collar, mild-to-moderate gliosis	Postmortem DBS brain bank study (8)
Fibrosis/collagen deposition	Rare but notable chronic reaction	Collagenous fibrosis, hemosiderin deposition, chronic inflammation	Postmortem case report (49)
Electrode-tissue interface remodeling	Dynamic biological-electrical interface	Changes in impedance, encapsulation tissue, peri-electrode space	Computational, clinical, and animal studies (54–57)
Active vs inactive contacts	Similar tissue response may occur around both	Thin glial capsule, foreign body reaction, local hemosiderin and gliosis	Postmortem STN-DBS analysis (50)

DBS, deep brain stimulation; T2/FLAIR, T2-weighted/fluid-attenuated inversion recovery imaging; MRI, magnetic resonance imaging; GFAP, glial fibrillary acidic protein; STN, subthalamic nucleus.

## 4 Stimulation-induced neuroimmune and neurometabolic modulation in PD

### 4.1 Central immune remodeling and glial responses

Electrical stimulation delivered by DBS may reshape the neuroimmune microenvironment through mechanisms that extend beyond the tissue reaction induced by electrode implantation. This distinction is essential because electrode insertion can itself produce microlesion effects, BBB disruption, local gliosis, and microglial recruitment, whereas active stimulation may additionally modulate neuronal firing, neurotransmitter release, glial phenotype, cytokine production, and network-level inflammatory tone. In PD, where neuroinflammation is increasingly recognized as a contributor to dopaminergic vulnerability, DBS-induced immune remodeling may represent a biologically meaningful component of its therapeutic action rather than a nonspecific consequence of surgery (12). The reported neuroimmune effects of DBS and the translational implications were listed in **Table 2**.

**Table 2:** Neuroimmune effects, neuroprotective mechanisms, and translational implications of DBS.

Domain	Main finding	Proposed mechanism	Evidence type	Translational implication
Central glial modulation in PD	STN-DBS may reduce glial activation and inflammatory signaling	Microglial and astrocytic phenotype remodeling; reduced cytokine expression	6-OHDA PD rat model and mechanistic reviews (21, 63, 69)	Supports DBS as a potential modulator of local neuroinflammation
Inflammasome activity	DBS may decrease inflammasome reactivity in the striatal microenvironment	Reduced inflammatory cascade and glial stress	6-OHDA PD rat model (21)	Provides a mechanistic link between stimulation and anti-inflammatory effects
Glutamate clearance	DBS may increase EAAT2 expression and reduce extracellular glutamate	Improved astrocytic glutamate uptake; reduced excitotoxic-inflammatory coupling	6-OHDA PD rat model (21)	Connects circuit modulation with neuroimmune protection
Peripheral immune changes	DBS may alter lymphocyte dynamics and Th17/Treg balance	Shift from pro-inflammatory to regulatory immune phenotype	Human cohort and α-synuclein PD animal model (20)	Suggests blood immune markers may reflect DBS-related biological effects
Cytokines and chemokines	DBS may influence TNF-α, RANTES, IL-1β, IL-6, and CXCL12	Modulation of inflammatory mediator expression	Clinical biomarker study and human CNS cell model (17, 22)	Candidate biomarkers for future longitudinal DBS studies
Neurotrophic signaling	STN-DBS may engage BDNF/TrkB pathways	Enhanced neuronal resilience and synaptic plasticity	Preclinical neuroprotection studies (68–71)	Supports neuroprotective potential, but clinical proof remains limited
α-synuclein-related pathology	DBS effects may depend on disease model and proteinopathy stage	Possible interaction with synucleinopathy and immune activation	Mixed preclinical evidence (65, 76)	Highlights need for α-syn-specific DBS models
Cross-disease neuroimmune effects	Stimulation can remodel immune responses in stroke, pain, and developmental immune models	Disease-specific glial and immune-state modulation	Stroke, neuropathic pain, and maternal immune activation models (78, 79, 84)	Suggests immune effects are context-dependent, not PD-specific
Biomarker development	TSPO-PET, cytokines, immune profiling, and single-cell omics may track DBS effects	Multimodal monitoring of neuroinflammation	PET imaging, immune profiling, and single-cell studies (90, 95, 98)	Enables biomarker-guided DBS trials
Adaptive/immune-informed DBS	Future DBS may integrate immune biomarkers with electrophysiological feedback	Closed-loop adjustment based on neural and biological states	Closed-loop DBS and machine-learning neuromodulation literature (97–99)	Provides a path toward precision neuromodulation

PD, Parkinson’s disease; DBS, deep brain stimulation; STN, subthalamic nucleus; 6-OHDA, 6-hydroxydopamine; EAAT2, excitatory amino acid transporter 2; Th17, T helper 17 cells; Treg, regulatory T cells; TNF-α, tumor necrosis factor-α; RANTES, regulated upon activation, normal T cell expressed and secreted; IL, interleukin; CXCL12, C-X-C motif chemokine ligand 12; CNS, central nervous system; BDNF, brain-derived neurotrophic factor; TrkB, tropomyosin receptor kinase B; TSPO-PET, translocator protein positron emission tomography; PET, positron emission tomography.

Microglia and astrocytes are central to this process. In PD, activated microglia can release TNF-α, IL-1β, IL-6, nitric oxide, prostaglandins, and reactive oxygen species, thereby amplifying oxidative stress and neuronal injury (13, 61, 62). Astrocytes, while essential for glutamate clearance, trophic support, and metabolic homeostasis, may also acquire reactive phenotypes that perpetuate inflammatory signaling and synaptic dysfunction (13, 61). T lymphocytes further interact with these glial responses; CD4+ T-cell infiltration contributes to dopaminergic neurodegeneration in experimental PD, and α-synuclein-specific T-cell responses have been identified in patients with PD (28, 30). Thus, stimulation-induced changes in glial and immune activity occur within a disease environment already primed toward inflammatory amplification.

Experimental evidence supports this view. In a 6-OHDA rat model, STN-DBS reduced striatal cytokine expression, decreased inflammasome reactivity, inhibited astrocytic morphological changes, and increased EAAT2 expression, suggesting a shift toward improved astrocytic glutamate handling and reduced inflammatory activation (21). Importantly, both microlesion and stimulation influenced some inflammatory markers, but active DBS produced additional effects on astrocytic morphology, inflammasome activity, and EAAT2, supporting the presence of stimulation-specific immune modulation (21). Human cellular models also indicate that clinically patterned electrical stimulation can directly affect inflammatory mediator expression. Kubelt et al. found that simulated DBS did not impair survival or proliferation of human astroglial cells, microglia, or differentiated neurons, but selectively increased CXCL12 in astrocytes and IL-1β in differentiated neuronal cells (22). These findings caution against describing DBS as uniformly anti-inflammatory.

Together, current evidence suggests that DBS induces context-dependent neuroimmune remodeling rather than simple immune suppression. Its effects depend on cell type, disease state, stimulation parameters, target anatomy, and the local neurochemical-metabolic environment. In PD, such remodeling may be especially relevant because glial activation, cytokine signaling, inflammasome activity, T-cell involvement, mitochondrial stress, and excitotoxic pressure converge on the vulnerable nigrostriatal system (12, 17, 20–22).

### 4.2 Suppression of neuroinflammation and excitotoxic-inflammatory coupling

PD pathology is not driven by inflammation alone. Inflammatory mediators interact with abnormal glutamatergic transmission, mitochondrial dysfunction, oxidative stress, and α-synuclein pathology. STN hyperactivity may increase downstream glutamatergic drive, and excessive extracellular glutamate can activate microglia and astrocytes, disrupt calcium homeostasis, and facilitate cytokine release. Therefore, DBS may attenuate neuroinflammation partly by restoring neurotransmitter balance and reducing excitotoxic pressure.

The study by Pinheiro Campos et al. provides particularly relevant mechanistic evidence. In a 6-OHDA PD rat model, STN-DBS reduced extracellular glutamate, decreased cytokine expression, inhibited inflammasome reactivity, and increased EAAT2 expression in the striatum (21). EAAT2 is primarily involved in astrocytic glutamate uptake. Thus, its upregulation suggests that DBS may improve astrocytic homeostatic function while reducing glutamate-driven inflammatory activation. This is important because astrocytes are not passive markers of injury. They actively regulate synaptic extracellular glutamate, metabolic support, and inflammatory tone.

The metabolic dimension of this mechanism is important. Astrocytic glutamate transport is coupled to energy-demanding ion gradients, glutamate-glutamine cycling, lactate production and release, and antioxidant support. Therefore, astrocytes occupy a central intersection among inflammation, excitotoxicity, and energy metabolism (41, 42). By improving astrocytic glutamate buffering, DBS may indirectly reduce microglial activation, limit cytokine release, and lower neuronal metabolic stress. This interpretation remains mechanistic rather than definitive, but it provides a biologically coherent link between stimulation-induced changes in extracellular glutamate, glial phenotype, and metabolic resilience (21, 41, 42). Thus, the anti-inflammatory and neurometabolic effects of DBS should not be viewed as separate outcomes, but as potentially coupled processes mediated through astrocytic glutamate handling, mitochondrial stress responses, and microglia-astrocyte communication.

Clinical biomarker data are consistent with this anti-inflammatory interpretation, although causality remains incompletely established. Chang et al. measured serum RANTES and TNF-α in preoperative DBS PD patients, postoperative DBS PD patients, and controls. RANTES correlated with Hoehn-Yahr stage and motor severity in PD patients, and the authors proposed that DBS may improve the inflammatory environment associated with PD (17). Because RANTES participates in recruitment of monocytes, macrophages, and T lymphocytes, this finding links DBS outcomes to immune-cell trafficking and inflammatory signaling. More broadly, recent reviews have emphasized that DBS may influence cytokine release, glial activity, oxidative stress, synaptic function, and neuroprotective pathways (3, 4, 63, 64). However, postoperative inflammatory changes may reflect electrical stimulation, microlesion effects, motor improvement, or systemic stress reduction. Therefore, DBS should not be framed simply as an anti-inflammatory intervention.

### 4.3 Peripheral immune changes and brain-peripheral crosstalk

Although DBS is applied locally within the brain, its immune consequences may extend beyond the stimulated nucleus. PD is increasingly understood as a systemic disorder. Peripheral immune cells may influence CNS inflammation through cytokine signaling, BBB interactions, and antigen-specific responses. CD4+ lymphocytes contribute to dopaminergic neurodegeneration in experimental PD, and α-synuclein-specific T-cell responses have been demonstrated in patients (28, 30). Regulatory T cells, conversely, can attenuate Th17-mediated nigrostriatal degeneration in preclinical models (31). These findings provide a mechanistic rationale for examining whether DBS affects peripheral immune balance.

The most direct evidence comes from McFleder et al., who reported that DBS may halt PD-related immune dysregulation in both brain and peripheral blood (20). Using longitudinal data from the Parkinson’s Progression Markers Initiative, they found that circulating lymphocytes progressively declined in PD and that this decline predicted future motor progression. DBS appeared to halt this progressive lymphocyte decline. In a second cohort, DBS was associated with a shift from pro-inflammatory CD4+ Th17 cells toward anti-inflammatory CD4+ regulatory T cells (20). This is highly relevant because Th17 responses are associated with inflammatory tissue injury, whereas regulatory T cells restrain excessive immune activation and may support immune tolerance. The same study used RNA sequencing and immunohistochemistry in an A53T α-synuclein rat model and found that DBS decreased neuroinflammation in the brain (20). This combined human and animal evidence suggests that DBS may influence immune networks across central and peripheral compartments. It also raises the possibility that peripheral immune markers could serve as accessible biomarkers for DBS-related biological effects.

Nevertheless, several questions remain unresolved. Peripheral immune changes after DBS may arise from direct neuromodulation of autonomic, hypothalamic, or neuroendocrine circuits, but they may also reflect indirect effects of improved mobility, better sleep, or changes in dopaminergic medication. Moreover, the direction of immune modulation may differ by disease stage, stimulation target, baseline inflammatory phenotype, and stimulation parameters. Therefore, longitudinal immune profiling and clinical outcomes should be integrated to clarify whether immune changes precede motor improvement, accompany it, or emerge as downstream consequences of improved network function.

### 4.4 Neurometabolic remodeling

DBS may induce neurometabolic remodeling through its effects on neuronal activity, glial state, and distributed basal ganglia-thalamo-cortical networks. Because neural activity is tightly coupled to energy demand, stimulation-induced changes in firing patterns, synaptic transmission, and network synchronization are expected to influence regional glucose utilization and metabolic network topology. Molecular imaging studies support this network-level view. A systematic review of molecular brain imaging studies concluded that DBS can produce measurable changes in neurotransmitter systems, regional metabolism, and distributed network activity rather than acting only at the electrode contact (8). In PD, FDG-PET studies suggest that STN-DBS can reshape cerebral glucose metabolism and PD-related metabolic network expression. Ge et al. reported that PD-related metabolic covariance pattern expression decreased after STN-DBS and generally paralleled motor improvement, supporting the use of metabolic network expression as an objective biomarker for DBS monitoring (7). Lee et al. found that preoperative glucose metabolic patterns were associated with the potential for motor improvement after DBS, suggesting that baseline network metabolic state may influence clinical responsiveness (9). Other PET studies have reported changes in vesicular monoamine transporter binding, glucose metabolism, DAT distribution, depressive symptoms, and cortical metabolic patterns after STN-DBS (65–67).

These findings indicate that DBS-induced metabolic effects are not confined to peri-electrode tissue. Instead, stimulation of a basal ganglia node can reorganize distributed motor, associative, and limbic networks. However, regional increases or decreases in FDG uptake should not be interpreted simplistically as neuroprotection or neurotoxicity. Therefore, FDG-PET findings should be interpreted as markers of stimulation-related network reorganization rather than direct surrogates of neuronal survival, neuronal injury, or disease modification. Such changes may reflect reduced pathological oscillatory activity, altered synaptic output, compensatory network recruitment, motor improvement, medication adjustment, or differences in stimulation state at the time of imaging. Thus, metabolic imaging provides an important systems-level readout of DBS action, but it should be integrated with electrophysiology, medication state, clinical outcomes, and longitudinal biomarkers before being used to infer disease modification (7–9, 66–68).

At the cellular level, DBS may also influence mitochondrial and redox biology. A recent translational study provides direct support for a mitochondrial-redox dimension of STN-DBS. Chen et al. reported that STN-DBS alleviated oxidative stress via mitophagy-related mechanisms in PD models and found changes in antioxidant enzymes in patients after surgery (23). In experimental systems, STN stimulation was associated with increased mitophagy, improved mitochondrial fission-fusion balance, enhanced antioxidant defenses, reduced release of mitochondrial apoptogenic factors, and dopaminergic neuroprotection. Pharmacological interference with mTOR signaling attenuated these effects (23). These findings identify mitochondrial quality control as a candidate pathway through which DBS may intersect with neuroprotection; however, the direct relevance of mTOR-autophagy regulation to human PD-DBS remains hypothesis-generating. Finally, neurotrophic signaling may further connect stimulation to metabolic resilience. STN-DBS has been reported to employ TrkB signaling for neuroprotection and functional restoration, increase BDNF in the nigrostriatal system and primary motor cortex, and partially restore disrupted striatal afferent BDNF in synucleinopathy models (69–71). BDNF/TrkB signaling can influence synaptic plasticity, neuronal survival, mitochondrial function, and cellular stress responses, but the extent to which these effects mediate DBS-induced metabolic remodeling in human PD remains uncertain. BDNF/TrkB signaling and mTOR-autophagy were illustrated in **Figure 3**.

**Figure 3:**
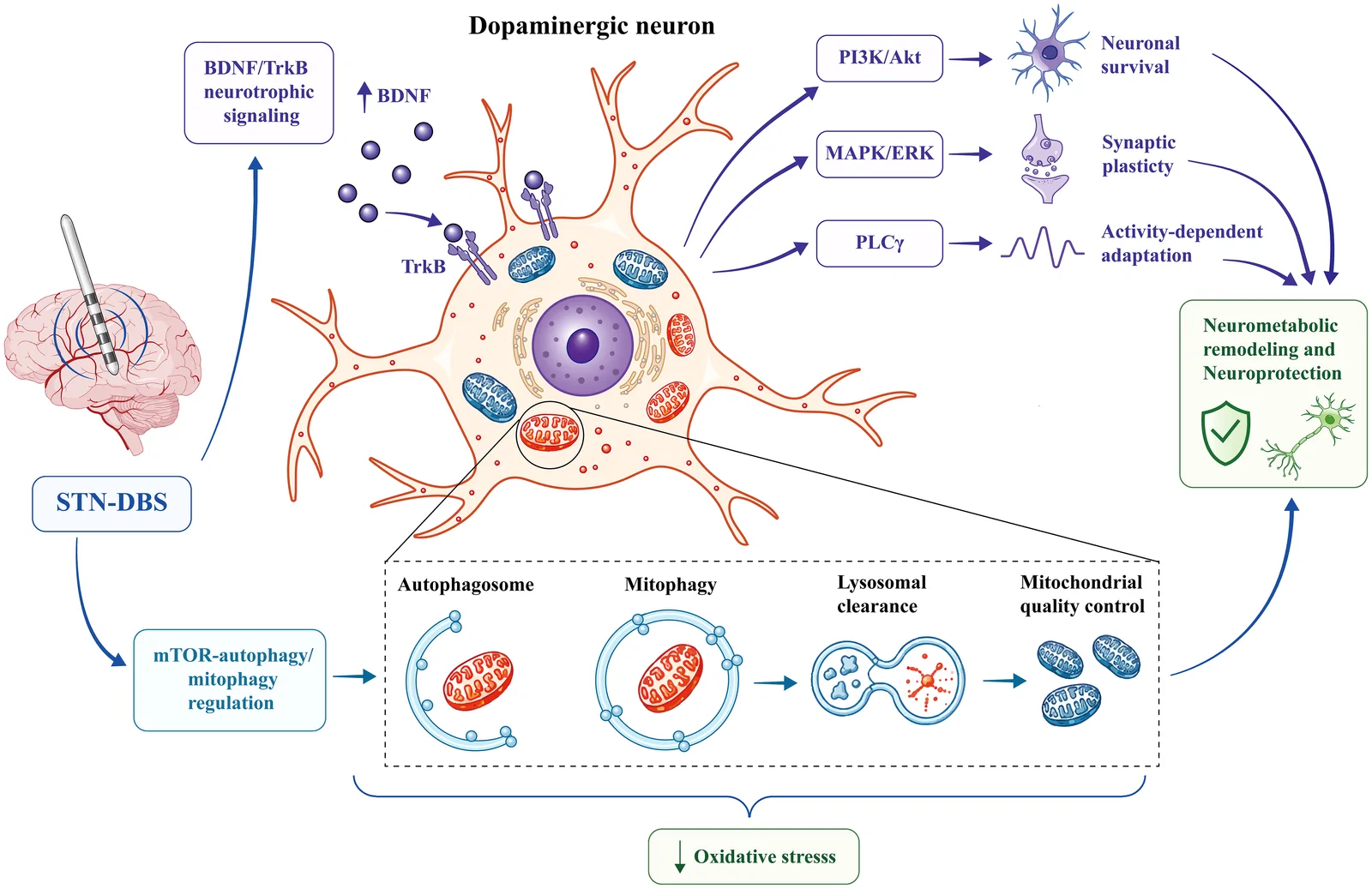
DBS-induced neurometabolic remodeling through mTOR-autophagy and BDNF/TrkB signaling. This schematic illustrates two proposed cellular pathways linking STN-DBS to dopaminergic neuronal resilience in PD. STN-DBS may enhance BDNF/TrkB signaling, activating downstream PI3K/Akt, MAPK/ERK, and PLCγ pathways to support neuronal survival, synaptic plasticity, and stress adaptation. In parallel, DBS may regulate mTOR-associated autophagy and mitophagy processes, promoting lysosomal clearance of damaged mitochondria and improving mitochondrial quality control, thereby reducing oxidative stress. These convergent mechanisms may contribute to neurometabolic remodeling and potential dopaminergic neuroprotection.

## 5 Neuroprotective potential of DBS in PD

The possibility that DBS may exert neuroprotective or disease-modifying effects in PD remains one of the most intriguing but unresolved questions in the field. Clinically, DBS is established as a symptomatic therapy that improves motor fluctuations, tremor, rigidity, and dyskinesia in appropriately selected patients. However, symptomatic improvement should not be equated with neuroprotection. A true neuroprotective effect would require evidence that DBS slows dopaminergic neuronal loss, preserves nigrostriatal projections, modifies α-synuclein pathology, or delays clinically meaningful disease progression independently of symptomatic masking (64, 72–74).

Several mechanisms make DBS biologically plausible as a neuroprotective intervention. At the circuit level, STN-DBS may reduce pathological basal ganglia activity and modify excessive glutamatergic output, thereby limiting excitotoxic stress on downstream structures (3, 4). At the cellular level, DBS may influence synaptic plasticity, mitochondrial function, neurotrophic signaling, glial reactivity, and inflammatory mediators (4, 64, 75). These mechanisms are not mutually exclusive. Indeed, neuroprotection in PD would likely require coordinated effects on mitochondrial-redox stress, astrocytic metabolic support, excitotoxicity, inflammation, and proteinopathy rather than a single pathway. These mechanisms should be interpreted according to evidence level. some are supported by direct DBS-related experimental data, whereas others remain biologically plausible candidate mechanisms that require validation in PD-DBS cohorts.

Most direct experimental support for candidate neuroprotective mechanisms comes from preclinical studies, whereas clinical evidence for true disease modification in PD-DBS remains insufficient. STN-DBS has been shown to engage brain-derived neurotrophic factor (BDNF)-related signaling pathways, particularly TrkB signaling, which may contribute to both functional restoration and preservation of nigrostriatal integrity (69). Earlier experimental work also reported that STN stimulation increased BDNF in the nigrostriatal system and primary motor cortex, suggesting that stimulation may recruit endogenous trophic mechanisms (71). In models involving synucleinopathy, striatal afferent BDNF signaling appears disrupted and may be partially restored by STN-DBS (70). These findings support a model in which DBS does more than suppress abnormal firing; it may alter molecular programs relevant to neuronal resilience.

However, the preclinical literature is not uniformly positive. In a nigrostriatal α-synuclein overexpression model, STN-DBS did not modify functional deficits or axonopathy, indicating that stimulation may fail to rescue established proteinopathy-driven degeneration under certain conditions (76). Similarly, STN-DBS did not alter growth factor expression in a rat model of stable dopaminergic deficiency, suggesting that neurotrophic effects may depend on model type, lesion stage, stimulation duration, and residual circuit integrity (77). These negative or neutral findings are important because they prevent overgeneralization from selected toxin-based studies.

Neuroimmune modulation may be one route through which DBS contributes to neuronal protection. In a 6-OHDA rat model, STN stimulation reduced striatal cytokine expression, decreased inflammasome reactivity, improved glutamate handling, and increased EAAT2 expression, linking anti-inflammatory effects with reduced excitotoxic stress (21). In patients and experimental models, DBS has also been associated with changes in inflammatory markers and immune-cell profiles, including evidence that DBS may halt PD-related immune dysregulation in brain and peripheral blood (17, 20). Neurometabolic evidence further supports this integrative framework. STN-DBS has been reported to alleviate oxidative stress through mitophagy-related mechanisms in PD models, with associated changes in antioxidant enzymes in patients (23). Molecular imaging studies also show that DBS can reshape distributed metabolic networks, and longitudinal FDG-PET-derived metabolic network measures may track DBS-related clinical changes (7, 8). These findings suggest that neuroprotection, if present, may emerge from convergence among circuit modulation, immune recalibration, mitochondrial quality control, and network-level metabolic reorganization. Nevertheless, this remains a candidate framework rather than proof of disease modification.

Clinical evidence for disease modification remains more limited. Long-term DBS studies show sustained motor benefit for selected symptoms, but axial symptoms, cognition, speech, gait, and non-motor features may continue to progress. This pattern suggests that DBS modifies circuit output more reliably than it modifies the underlying multisystem neurodegenerative process (72, 73). Comparative analyses of surgical approaches to disease modification likewise emphasize that current clinical data do not yet establish DBS as a proven disease-modifying therapy (74). Therefore, DBS should be described as having neuroprotective potential, not confirmed neuroprotective efficacy.

## 6 Neuroimmune effects beyond PD-DBS and translational challenges

### 6.1 Neuroimmune effects beyond PD-DBS

Although this review focuses on PD, DBS-related neuroimmune modulation is not restricted to nigrostriatal disease. Evidence from stroke, neuropathic pain, Alzheimer’s disease (AD), psychiatric models, and peripheral neuromodulation suggests that electrical stimulation can alter glial activity, inflammatory signaling, immune-cell phenotypes, and repair-associated programs in a disease- and target-dependent manner.

In ischemic stroke, hippocampal DBS improved motor and behavioral outcomes and reshaped microglial subpopulations at single-cell resolution. Specific microglial subsets associated with inflammation and angiogenesis were modified by stimulation, suggesting that DBS may promote repair-oriented immune remodeling rather than simply suppress inflammation (65). In neuropathic pain, motor cortex stimulation reduced spinal astrocyte and microglial activation, decreased TNF-α and IL-1β staining, and modulated cannabinoid and purinergic signaling; persistent neuroimmune abnormalities were associated with stimulation refractoriness (78). These findings support the concept that glial-inflammatory state may influence responsiveness to neuromodulation.

Other disease models further expand this view. DBS during early adolescence prevented microglial alterations in a maternal immune activation model (schizophrenia), indicating that stimulation can interact with neurodevelopmental immune programming (79). In Alzheimer’s disease, fornix and nucleus basalis DBS have been explored for effects on memory networks, metabolism, synaptic plasticity, and possibly neurodegenerative progression, although the immune mechanisms remain less defined than in PD or stroke models (72, 80, 81). Peripheral neuromodulation also provides relevant evidence: auricular and cervical vagus nerve stimulation reduced neuroinflammation and improved motor behavior in PD models (82, 83). Together, these studies suggest that immune modulation may be a shared but context-dependent feature of electrical neuromodulation. Neuroimmune effects of neuromodulation beyond PD-DBS were summarized in **Table 3**.

**Table 3:** Neuroimmune effects of neuromodulation beyond PD-DBS.

Disease	Stimulation target	Neuroimmune findings	Interpretation for DBS neuroimmunology
Ischemic stroke (65)	Hippocampal DBS	Improved neurobehavioral outcomes and promoted neurorepair; single-cell analysis showed remodeling of neuronal, astrocytic, oligodendroglial, and microglial populations, including microglial subsets linked to immune inflammation and angiogenesis.	Supports the concept that DBS can redirect repair-associated immune programs rather than merely suppressing inflammation.
Neuropathic pain (78)	Motor cortex stimulation	Reduced spinal astrocyte and microglial activation, decreased TNF-α and IL-1β immunostaining, and modified cannabinoid and purinergic signaling in the dorsal horn; refractory animals retained neuroimmune abnormalities.	Suggests that glial-inflammatory normalization may contribute to therapeutic responsiveness and that immune state may shape neuromodulation outcomes.
Schizophrenia (79)	Medial prefrontal cortex DBS	Prevented microglial alterations induced by maternal immune activation and improved disease-relevant behavioral and circuit phenotypes in related work.	Shows that stimulation can interact with developmental neuroimmune programming, although direct translation to PD remains limited.
Alzheimer’s disease (72, 80, 81)	Fornix DBS and nucleus basalis of Meynert DBS	Fornix DBS studies primarily demonstrated effects on memory circuits and cerebral metabolism, whereas emerging nucleus basalis work has begun to link stimulation with possible anti-inflammatory mechanisms.	Provides a comparator proteinopathy platform for testing whether circuit stimulation can influence neuroinflammatory trajectories across neurodegenerative diseases.
Peripheral neuromodulation in PD models (82, 83)	Vagus nerve stimulation	Reduced neuroinflammation, exerted immune-regulatory effects, and improved behavioral or neuroprotective outcomes in 6-OHDA and related PD models.	Indicates that immune modulation may be a shared principle across invasive DBS and peripheral stimulation modalities, while anatomical routes and mechanisms differ.

PD, Parkinson’s disease; DBS, deep brain stimulation; TNF-α, tumor necrosis factor-α; IL-1β, interleukin-1β; 6-OHDA, 6-hydroxydopamine.

### 6.2 Translational barriers

Several barriers limit translation of DBS neuroimmune findings. First, immune changes after DBS may reflect electrode implantation, microlesion effects, active stimulation, improved motor function, altered sleep, or reduced systemic stress. Implantation alone can induce gliosis and inflammatory remodeling, so stimulation-specific effects must be separated from tissue-interface responses (4, 11).

Second, disease models vary substantially. Toxin-based PD models, α-synuclein models, stroke models, pain models, and AD models each produce distinct inflammatory environments. A stimulation protocol that reduces maladaptive microglial activation in one model may fail to modify proteinopathy or axonopathy in another. Therefore, cross-disease evidence should generate hypotheses rather than support a universal claim that DBS is anti-inflammatory.

Third, immune and metabolic biomarkers capture different biological levels and should not be interpreted interchangeably. FDG-PET reflects regional glucose utilization and network activity, whereas TSPO-PET is used as an *in vivo* proxy for neuroinflammatory signal, with important limitations related to ligand specificity and cellular interpretation (84, 85). Plasma metabolomics and lipidomics reflect systemic biochemical states that may be influenced by diet, medication, body weight, physical activity, sleep, and autonomic function, while CSF metabolites may be closer to CNS biology but remain affected by sampling timing and disease stage (86). Fluid biomarkers such as mitochondrial DNA, neurofilament light chain, and α-synuclein provide complementary information about mitochondrial injury, axonal damage, and synucleinopathy, but they do not measure the same process and should not be collapsed into a single category of “metabolic improvement” or “disease modification” (87–89).

### 6.3 Future directions

Future studies should move from descriptive associations toward temporally resolved and mechanistically interpretable biomarkers of DBS-induced neuroimmune-neurometabolic remodeling. A key priority is to distinguish implantation-related responses, stimulation-specific biological effects, medication-related changes, and secondary consequences of improved motor function. This requires longitudinal sampling before surgery, after electrode implantation, after stimulation initiation, after programming stabilization, and during long-term follow-up.

Imaging should link regional biology with circuit-level effects. TSPO-PET and related neuroinflammation imaging approaches may help estimate glial activation *in vivo*, although ligand specificity and cellular interpretation remain important limitations (84, 90). FDG-PET and metabolic brain network approaches can complement this by capturing regional glucose utilization and distributed network activity (8, 85). Rather than interpreting either modality alone, future DBS studies should examine whether inflammatory and metabolic imaging signals are temporally coupled, spatially dissociated, or differentially related to motor and non-motor outcomes.

Fluid biomarkers should be selected according to specific biological questions rather than collected as an undifferentiated panel. Neurofilament light chain may help estimate axonal injury and disease progression, whereas α-synuclein seed amplification assays may help stratify synucleinopathy biology and reduce cohort heterogeneity (87, 89). Metabolomics and lipidomics may be useful for assessing systemic or CSF biochemical states linked to energy metabolism, redox balance, and lipid remodeling (86). Mitochondrial DNA may be explored as a marker of mitochondrial injury or sterile inflammatory signaling, but its interpretation in DBS cohorts will require careful control for surgical timing, systemic stress, and disease stage (88). Recent analyses of PPMI data indicate that baseline α-synuclein seed amplification assay status does not reliably predict longitudinal motor, non-motor, cognitive, or dopamine transporter imaging progression in manifest PD (91). Similarly, NfL and GFAP may be informative markers of axonal or astroglial injury after DBS surgery, but available studies suggest that they often show transient postoperative increases related to surgical tissue injury and then return toward baseline over weeks to months (92–94). Therefore, these biomarkers should not be used in isolation as evidence of disease modification or immune-mediated neuroprotection. Future studies should combine progression-relevant clinical milestones, imaging markers, seed amplification assays, neuronal injury markers, glial markers, and immune profiling in longitudinal designs.

Cell-type-resolved methods will be necessary to identify which cellular compartments are remodeled by DBS. Single-cell and spatial transcriptomic studies of the human substantia nigra and midbrain have revealed PD-associated neuronal states and glial activation patterns, providing a framework for interpreting stimulation-related cellular responses (95, 96). Future studies should extend this approach to peri-electrode tissue, connected basal ganglia regions, cortical network nodes, and peripheral immune compartments when available.

Finally, adaptive DBS provides an opportunity to connect biological endpoints with stimulation control. Current adaptive systems mainly use electrophysiological biomarkers, especially local field potentials (97, 98). Future approaches could cautiously integrate immune profiles, metabolic biomarkers, wearables, and clinical outcomes to distinguish symptomatic circuit modulation from sustained neuroimmune-neurometabolic remodeling relevant to PD progression (99).

## 7 Conclusion

DBS in PD should be viewed as more than symptomatic circuit modulation. Electrode implantation and chronic stimulation interact with local tissue responses, immune signaling, and metabolic homeostasis. These effects provide plausible mechanisms for neuronal resilience and potential neuroprotection, but they do not yet prove disease modification. Future longitudinal and multimodal studies must distinguish stimulation-specific effects from surgery, medication changes, clinical improvement, and systemic factors. Only then can the field determine whether DBS meaningfully alters the neuroimmune-metabolic processes that drive PD progression.
